# Assignment-free chirality detection in unknown samples via microwave three-wave mixing

**DOI:** 10.1038/s42004-022-00641-3

**Published:** 2022-03-14

**Authors:** Greta Koumarianou, Irene Wang, Lincoln Satterthwaite, David Patterson

**Affiliations:** 1 Physics Department, University of California, Santa Barbara, Santa Barbara, CA USA; 2 Department of Chemistry and Biochemistry, University of California, Santa Barbara, Santa Barbara, CA USA

**Keywords:** Chemical physics, Circular dichroism

## Abstract

Straightforward identification of chiral molecules in multi-component mixtures of unknown composition is extremely challenging. Current spectrometric and chromatographic methods cannot unambiguously identify components while the state of the art spectroscopic methods are limited by the difficult and time-consuming task of spectral assignment. Here, we introduce a highly sensitive generalized version of microwave three-wave mixing that uses broad-spectrum fields to detect chiral molecules in enantiomeric excess without any prior chemical knowledge of the sample. This method does not require spectral assignment as a necessary step to extract information out of a spectrum. We demonstrate our method by recording three-wave mixing spectra of multi-component samples that provide direct evidence of enantiomeric excess. Our method opens up new capabilities in ultrasensitive phase-coherent spectroscopic detection that can be applied for chiral detection in real-life mixtures, raw products of chemical reactions and difficult to assign novel exotic species.

## Abstract (web summary)

Identification of chiral molecules in multi-component mixtures of unknown compositions remains a challenge. Here, the authors show that microwave three-wave mixing that uses broad-spectrum fields can detect chiral molecules in enantiomeric excess without prior chemical knowledge of the sample.

## Introduction

Many biomolecules, including DNA, proteins, and amino acids, are chiral, meaning they exist in two versions that are non-superimposable mirror images. Chirality is such a ubiquitous property in biology that more than 50 percent of active ingredients in pharmaceuticals are chiral^1^. Chiral molecules span other multi-billion dollar industries like the food industry, agriculture, and fragrances. In 2016, the first chiral molecule was detected in space^2^, re-sparking conversations on the implications of molecular chirality for the origins of life. However, currently established methods cannot determine enantiomeric excess, a signature of life, in complex raw samples like the ones collected from extraterrestrial environments.

Despite these broad applications, a general method for detecting and measuring enantiomeric excess remains elusive. While notable progress has been made towards the detection of slight enantiomeric excess on the 0.4% level^3^, detection of enantiomeric excess in unknown complex samples has proven challenging. Chromatography has long been the go-to method for enantiomeric analysis among synthetic chemists, however, as detection is based on chemical interactions, it cannot be generalized to unknown samples. Mass spectrometry and nuclear magnetic resonance (NMR) rely on chiral derivatization reagents and can be sensitive to contaminants^4–6^. For unknown multi-component mixtures, polarimetry can be inconclusive, as the calculation of specific rotation requires knowledge of concentration and it is often referenced to neat samples^7–9^.

Spectroscopic methods such as vibrational, photoelectron circular dichroism^10–14^, and microwave spectroscopy^15–23^ can be mixture compatible and provide highly accurate information on species identity. So far, these methods have been limited by spectral assignment; prior to any chirality experiment, the spectrum of the molecule had first to be collected and fully assigned. Considerable efforts have been made to automate and simplify spectral assignment^24–27^; nonetheless, it is still a difficult and time-consuming task conducted mainly by trained spectroscopists.

In this work, we demonstrate a generalized assignment-free version of microwave three-wave mixing (M3WM)^28–30^ that can identify chiral species in enantiomeric excess in unknown complex samples. We achieve this by exploiting our high sensitivity and employing broadband excitation pulses to search experimentally for transitions in a three-level system, along with the implementation of careful cancellation schemes to ensure that signals from species not in enantiomeric excess are subtracted. The resulting spectra, referred to here as “three-wave mixing spectra”, can provide direct proof on the existence of chiral species in enantiomeric excess and can be used for the study of previously hard-to-analyze samples: unassigned species and unknown complex mixtures. While the samples used in this work were not prepared by an outside team and were thus strictly speaking ‘known’ to our team, M3WM and proof of enantiomeric excess was demonstrated on these samples with no sample-dependent settings.

### Broadband three-wave mixing

**Fig. 1:**
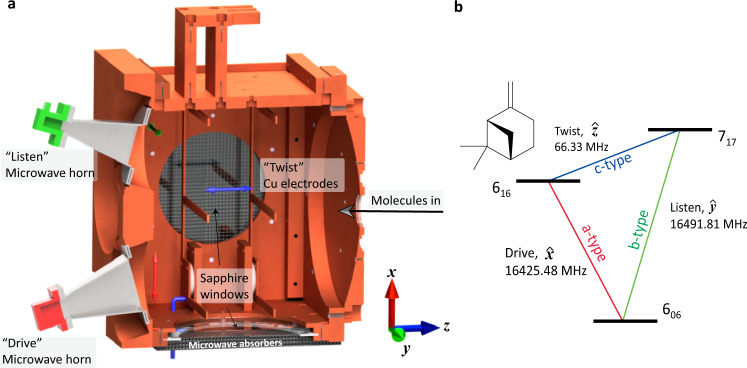
Three-wave mixing spectra in a buffer gas cell. **a** Cut-away of the buffer gas cell used for acquiring three-wave mixing spectra. Arrows colored in red, blue, and green indicate the polarization of the drive, twist, and listen pulses. The back and bottom side of the cell have sapphire windows covered on the outside with microwave absorbers. **b** A typical M3WM excitation scheme for *β*-pinene.The three transitions are polarized perpendicular to one another and form a triangle that consists of an a-type, a b-type, and c-type transition.

Our broadband assignment-free three-wave mixing uses broadband microwave and RF excitation combined with careful cancellation schemes. Polarization and phase controllability for broadband pulses are achieved with an updated experimental setup and microwave circuit (discussed more in detail under Methods). While knowing the exact phase of the relevant component of each chirp is challenging, the repeatability of this phase is excellent, as it must be in all chirp pulse microwave three-wave mixing experiments, and it can be accurately reversed by changing the phase of the signal coming from the arbitrary waveform generators. The resulting three-wave mixing spectra include numerous transitions from each chiral molecule that is present in enantiomeric excess. Each of those transitions stem from a M3WM excitation scheme, an example of which is shown in Fig. 1b. It is a three-level system of rotational energy levels that are connected via an *a*-type transition, a *b*-type transition, and a *c*-type transition, along each of three rotational axes. Two of these transitions are typically in the GHz frequency range, and the third transition is on the order of 100 MHz. The stimulated microwave transition (with frequency ~ 10 GHz) is referred to as the “drive” transition and the stimulated RF transition (with frequency ~ 100 MHz) is referred to as the “twist” transition^28^. The molecular ensemble emits radiation coherently at the “listen” frequency, which is detected and plotted as a spectrum. Previous M3WM experiments were limited to assigned, known species, required prior knowledge of the transitions, and reported enantiomeric excess based on a single excitation scheme like the one shown in Fig. 1b^28,29,31–33^.

## Results

### M3WM spectra of chiral and non-chiral species

**Fig. 2:**
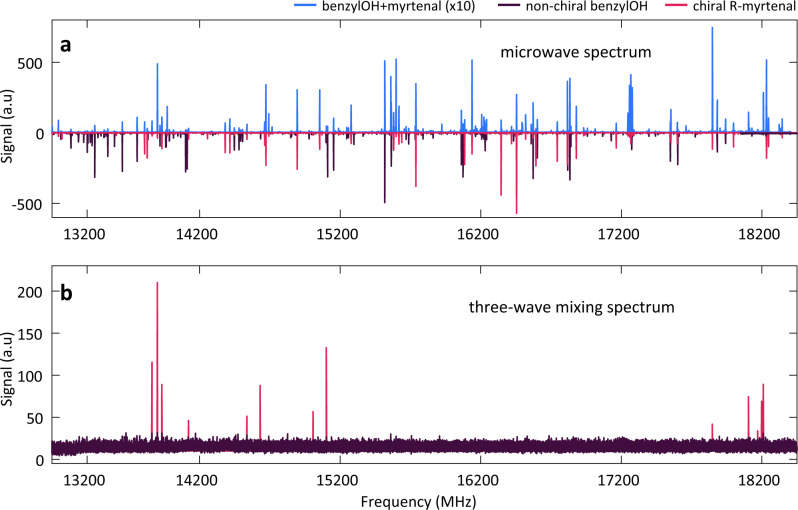
Comparison between microwave spectra and three-wave mixing spectra. **a** Top in light blue, microwave spectrum of the mixture of (R)-myrtenal (chiral) and benzyl alcohol (non-chiral). Amplitudes are multiplied by a factor of 10 for better visibility. Inverted, in dark purple and pink, the microwave spectra of the individual species, benzyl alcohol and myrtenal respectively. **b** Three-wave mixing spectrum of the mixture of (R)-myrtenal and benzyl alcohol. Each transition belongs to a chiral species in enantiomeric excess. In pink, only transitions from enantiopure (R)-myrtenal survive cancellation.

Figure 2 highlights the difference between microwave spectra and three-wave mixing spectra. Figure 2a shows the microwave spectrum of a mixture of a chiral molecule ((R)-myrtenal) and a non-chiral molecule (benzyl alcohol), compared to spectra of the individual components. In this frequency range, numerous rotational transitions from both species are present. Figure 2b shows the three-wave mixing spectrum of the same mixture, taken under similar conditions. Three-wave mixing spectra are non-zero only for chiral molecules in enantiomeric excess. Only transitions from enantiopure R-myrtenal are observed, as transitions from the non-chiral benzyl alcohol do not survive subtraction. It is noticeable that the transitions in the three-wave mixing spectrum are significantly fewer in number than the lines in the microwave spectrum of myrtenal, which is expected as not all transitions can participate in a M3WM chirality detection scheme, as the one shown in Fig. 1b.

Both spectra were recorded at 7 K, from 13,000–18,250 MHz and a He buffer gas flow of 10 sccm. The M3WM spectrum is assembled from 485 individual spectral segments, with 22.5 MHz local oscillator steps between them and acquired with a 35 MHz broadband drive pulse and an RF pulse with a range of 60–105 MHz for a total integration time of 3.5 h. For a list of the M3WM transitions see Supplementary Table 1.

### M3WM spectrum of racemic samples

**Fig. 3:**
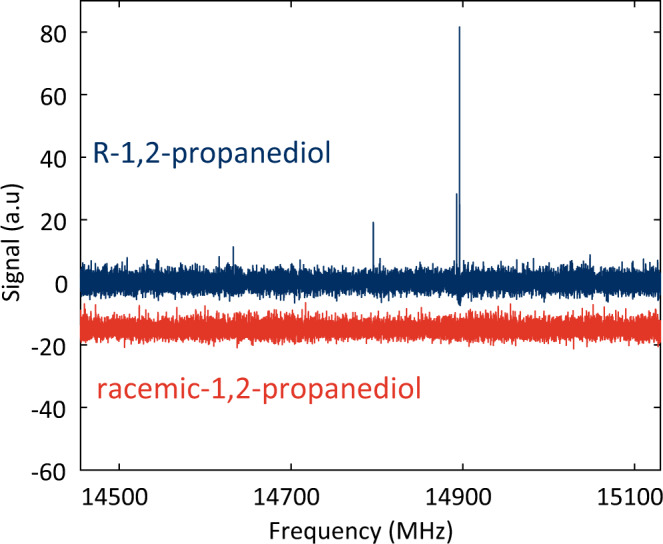
Three-wave mixing spectra of enantiopure vs racemic samples. (blue) Three-wave mixing spectrum of enantiopure (R)-1,2-propanediol. The three noticeable three-wave mixing signals belong to the two lowest energy conformers of 1,2-propanediol. (red) Three-wave mixing spectrum of racemic 1,2-propanediol. An offset of −15 (a.u) on the y-axis has been added to the racemic spectrum for clarity to show that no signal survives subtraction as expected.

M3WM spectra are designed to detect species in enantiomeric excess. Figure 3 shows the comparison between the M3WM spectrum of an enantiopure sample of (R)-1,2-propanediol, shown in blue, plotted against the M3WM spectrum of a racemic sample of 1,2-propanediol, in red. The M3WM signal of enantiopure (R)-1,2-propanediol shows three noticeable signals corresponding to the lowest and the third-lowest in energy (0.88 kJ/mol) conformer^34^. In contrast, these three-wave mixing signals are not present in the spectrum of the racemic sample, in red, which has been shifted by −15 (a.u) on the y-axis for clarity. The details of the methods used to eliminate non-chiral signals are described in detail below.

Both spectra were recorded in the range between 14,500–5100 MHz with a He buffer gas flow of 10 sccm, at 7 K. Each spectrum is assembled from 72 individual spectral segments, with 22.5 MHz local oscillator steps between them and acquired with a 35 MHz wide drive pulse and a twist pulse range of 80–105 MHz for a total integration time of 1 h. For a list of the M3WM transitions see Supplementary Table 2.

### M3WM spectrum of multi-component mixtures

**Fig. 4:**
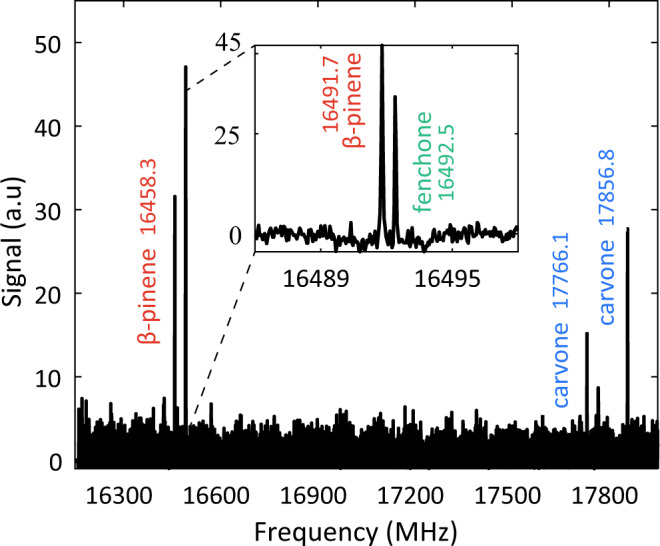
Three-wave mixing spectra for a mixture of chiral terpenes. Enantiopure (-)-*β*-pinene, (R)-fenchone, and (R)-carvone were identified within the mixture with a narrow scan between 16,300–18,000 MHz and two twist ranges from 65 to 85 MHz and 85 to 105 MHz.The inset zooms into the transition around 16,492 MHz which consists of two M3WM signals: a *β*-pinene M3WM signal at 16,491.7 MHz and one from fenchone at 16,492.5 MHz.

Three-wave mixing spectra can provide useful chirality information of multi-component mixtures without any prior chemical processing, separation, or spectral assignment. This capability is relevant to asymmetric synthesis and chemical analysis of complex real-life samples. In Fig. 4, we show the three-wave mixing spectrum for a mixture of terpenes. Terpenes are naturally occurring chiral building blocks that have been used for decades as starting materials for the synthesis of natural products and active ingredients in pharmaceuticals, due to their abundance and low cost^35–37^. All transitions in Fig. 4 belong to enantiopure (-)-*β*-pinene, (R)-fenchone, and (R)-carvone. The inset zooms into the transition around 16,492 MHz which consists of two separate M3WM signals: a *β*-pinene M3WM signal at 16,491.7 MHz and a second one from fenchone at 16,492.5 MHz. For such mixtures, even polarimetry measurements can be inconclusive, as the sum of the angles for multiple components can cancel each other out. Equal amounts of neat (-)-*β*-pinene, (S)-carvone, and (R)-fenchone would have a total specific rotation ${[a]}_{20}^{D}$ of +7^∘^, the sum of each component, which carries significantly less chemical information than a spectrum. In contrast, the three-wave mixing spectrum of such mixture, as seen in Fig. 4, shows distinct transitions for each separate species. If microwave spectra of the species are available, even if unassigned, then no additional measurements are required to determine the exact identity of the species. For readily available chiral building blocks like the ones used here, species were easily and accurately identified.

The spectrum of the mixture was recorded in the range between 16,200–18,000 MHz with a He buffer gas flow of 10 sccm, at 7 K. Each of the 150 spectral segments was recorded with a drive pulse of 35 MHz bandwidth. The total acquisition time was 2 h. Two separate twist ranges of 65–85 MHz and 85–105 MHz were used for increased RF power to assure transitions of less polar species are sufficiently driven. For a list of the M3WM transitions see Supplementary Table 3.

## Discussion

Three-wave mixing spectra can be recorded for any sample that contains molecules that are vaporizable and have non-zero electric dipole moments across all rotational axes. Microwave spectroscopy is mixture, solvent, isomer, and isotopologue compatible meaning that no chemical processing is necessary in most cases prior to analysis^30,38,39^. Chiral information can be extracted on-the-spot as only transitions from species that are chiral and in enantiomeric excess survive cancellation. As shown in Figs. 2, 3 signals from racemic samples or non-chiral molecules average to zero.

A promising application would be the direct chiral detection of the raw constituents of one-pot asymmetric synthesis reactions^40,41^. Inside this flask, there are reactants, solvents, products, by-products, and catalysts. Even though large polyatomic molecules like catalysts cannot be easily seen, a comparison between the three-wave mixing spectrum before and after the reaction can identify any new chiral products, in enantiomeric excess produced, as in Fig. 4. Even molecules very similar in structure like terpenes can be unambiguously identified with microwave spectroscopy. Since any separation or purification of the sample is unnecessary for analysis, our method can act as a tool for the general search of chiral catalysts. Unlike polarimetry, once the spectra are acquired the exact transitions can be used to unambiguously identify the species produced.

Microwave three-wave mixing works best for strongly polar molecules as the matrix elements for rotational transitions depend linearly on the magnitude of the dipole moments across the A, B, and C rotational axes^28^. In this work, beta-pinene with dipole moments of ∣*μ*_*a*_∣ = 0.43, ∣*μ*_*b*_∣ = 0.58, ∣*μ*_*c*_∣ = 0.11 Debye was the least polar molecule under study^42^. Even though enantiopure samples were used for all experiments, three-wave mixing signals scale linearly with enantiomeric excess (ee), thus signals from species of enantiopurity above 30 percent should be sufficiently above the noise level to be detected. This could be improved with straightforward electronics updates. The determination of the exact percentage of enantiomeric excess and absolute configuration from microwave three-wave mixing spectra could be performed similarly to other M3WM experiments^31^ given that there are available samples of known enantiomeric excess for calibration. Without such a calibration sample, the method cannot determine the absolute configuration—that is, whether *R*− or *S*− is in excess—as this relies on absolute knowledge of the dipole moments for each enantiomer, which is not defined for an unknown sample^43^.

An important parameter of the experiment is the frequency range of the twist pulse. We know from experience that most molecules display transitions between 60 and 110 MHz so we chose to use this range for the “twist" pulse during all data acquisition. However, for a more complete analysis of unknown samples additional frequency ranges can be easily explored. We have encountered no chiral molecule without three-wave mixing transitions with a twist between 25 and 250 MHz, which is the range of our current RF amplifier: our method is thus expected to detect any common vaporizable small chiral molecule. Additionally, as molecules grow in size, their microwave spectra get more congested and they should typically exhibit richer M3WM spectra.

Three-wave mixing spectra of unknown samples are useful as preliminary scans for chiral species in enantiomeric excess. Given that the method does not require prior chemical knowledge of the sample for determination of the rotational transitions or any optimization for probing different species (as shown in Fig. 4), it can be directly applied to unknown samples. However, further analysis is needed for identifying each species of an unknown mixture. To determine the identity of the species one needs to search for the transitions in available spectral libraries like splatalogue^44^, CDMS^45^, or published experimental and calculated spectra. For more exotic species, it is possible to perform the experiment in reverse, going from broadband fields to resonant to identify all transitions of the three-level system. Then, double resonance experiments similar to the one performed by Martin-Drumel et al.^46^ can be conducted to determine the rotational constants and the structure of the unidentified species.

In summary, we have introduced a generalized version of M3WM that includes the capability of acquiring microwave three-wave mixing spectra in unassigned samples. M3WM spectra can provide direct evidence on enantiomeric excess on the spot without the need for prior spectral assignment via the combination of broadband excitation and careful signal cancellation. Our new method can be applied to particularly hard-to-analyze samples like unknown multi-component mixtures and hard-to-assign species and provides new methods for ultrasensitive phase-coherent spectroscopic detection.

## Methods

### Experimental setup

The main components of the buffer gas cell apparatus have been described in detail elsewhere^47^. Molecules flow continuously through a copper tube heated at 40 ^∘^C into the buffer gas cell held at 5–7 K. A schematic of the apparatus is shown in Fig. 1a. Cold He buffer gas flows continuously into the cell at a typical flow rate of 10 standard cubic centimeters per minute (sccm). Microwave horns are oriented with polarizations of $\hat{x}$ and $\hat{y}$ for excitation and detection, respectively. Two equally spaced copper electrodes are attached to the cell through 1" sapphire insulators to produce an electric field in the $\hat{z}$ direction. As in traditional M3WM, the “drive" and “listen" microwave horns are placed at 90^∘^. For additional polarization control while maintaining the cold environment inside the cell, sapphire windows (4 inches diameter) were added on two sides of the cell and microwave absorber foam was placed on the outside, as shown in Fig. 1a. We observed that covering the inside of the buffer gas cell with microwave absorber significantly increased the gas temperature.

The sample input consists of three main parts: a copper tube, a diaphragm valve, and a nipple loosely packed with glasswool. Depositing the sample on glasswool results in even evaporation and significantly reduces signal fluctuations over time, leading to highly repeatable measurements.

### Elimination of non-chiral signals

The most vital part of the experiment is to ensure that all signals stem from chiral species by successfully eliminating all non-chiral signals. We used three different methods to do so: (a) polarization control as described above, (b) fast subtractions, (c) an updated microwave circuit design which rapidly and simultaneously changes the sign of the “drive" and “twist" pulses ensuring phase controllability and accurate reversibility for broadband chirps.

Fast subtractions: A key component of the success of non-linear microwave spectroscopy in a buffer gas cell is its high spectral acquisition velocity^47^. Each data point of the three-wave mixing spectrum consists of 2.5 × 10^6^ averages. The calm, controlled environment of the buffer gas cell enables careful subtractions between measurements of opposite twist phase every few hundreds of μs for each data point of the spectrum. This is important since we noticed that any “asymmetries" in the electronics or the data acquisition process can cause non-chiral signals to leak through.

To solve this issue, we used a two-channel arbitrary waveform generator with very low time jitter (Siglent SDG6052X) to generate the “drive" and “twist" pulse. The timing window between each measurement and each experimental cycle was long enough (80 μs) to prevent any signal cross-talk between measurements. A 9400 series Quantum Composer was also used to precisely control the timing between the two chirp pulses of each experimental cycle to ensure careful subtraction. It is not clear that a similar experiment could be conducted in an apparatus with pulsed valves where shot-to-shot variability is often significant.

**Fig. 5:**
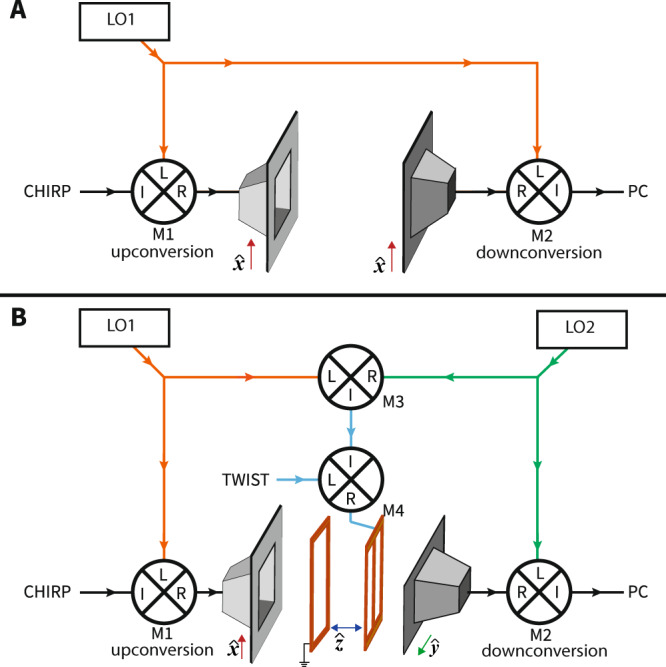
Comparison of microwave circuit design. **A** Schematic of the standard microwave circuit for microwave spectroscopy. **B** Schematic of the new circuit design for acquiring three-wave mixing spectra. The twist electrodes have been added for clarity. The upconversion and downconversion step are done with different local oscillators LO1 and LO2. Their beat note, 2 KHz in this work, is mixed with the twist to eliminate all non-M3WM signals.

Updated microwave design: An updated microwave circuit design ensures high phase coherence between the twist and drive pulses by mixing the twist pulse with the beat note between the upconversion and downconversion steps. Figure 5 shows a comparison between the conventional circuit for microwave spectroscopy and the updated design. In the new design, two different local oscillators, LO1 and LO2, are used for the upconversion and the downconversion step and their beat frequency is mixed with the twist pulse. Specifically, mixer (M3) was added to the circuit taking LO1 and LO2 as inputs (the frequency difference between them was set to 2 KHz). This beat note is AC-coupled and amplified, then fed into mixer (M4) where it is combined with the twist pulse. The offset local oscillators cause any 1D (non-chiral) signals to alternate phase with the 2 KHz beat note between the two local oscillators, and thus average to zero. The phase of the twist also alternates phase with the 2 KHz beat note between the two local oscillators, and so the M3WM signal survives and averages to a non-zero value. This signal is recorded alternatively with a generated twist phase of *ϕ* = 0 and a twist phase of *ϕ* = *π*, and signals from these two configurations are further subtracted before the spectrum is assembled. This final step removes small (< 30 dB) bleedthrough of 1D signals resulting from imperfect mixing in the twist generation (M4). The new circuit design should improve the statistics of enantiomeric excess determination for single-frequency M3WM experiments as well.

### Data acquisition

All spectra were collected with similar conditions to demonstrate the applicability of the method to a wide variety of species without selective optimization. A 4 μs long 35 MHz broadband microwave pulse is used as the “drive" pulse followed by 2 μs long RF twist pulse with a frequency chirp of 60–105 MHz. The “twist" pulse is overlapped with the drive pulse by 1 μs. The resultant coherent molecular signal (or “free induction decay" (FID)) following the double excitation is collected by a second orthogonally polarized horn and digitized to form the spectra measured such as in Fig. 2b.

Three-wave mixing spectra combine a non-linear detection method (M3WM) with broadband excitation. Since we necessarily don’t know transition dipole moments for unknown species, we operate at a pulse strength which is significantly underpowered for typical transitions. This results in typical signals that are 2–10 times weaker than typical M3WM signals under conditions optimized for maximum signal. These conditions were chosen to provide observable signal while not overdriving transitions, which would typically lead to larger false positives from non-chiral spectral lines, for more technical details see Supplementary Note.

### Chemicals

Commercial (R)-(-)-1,2 -propanediol (96% purity), anhydrous benzyl alcohol (99.8% purity), (1R)-(-)-myrtenal (98% purity), (1R)-(-)-fenchone (98% purity), (R)-(-)-carvone (98% purity), (-)-*β*-pinene (99% purity) were purchased from Sigma-Aldrich.

## Supplementary information

Supplementary Information

## Data Availability

The data that support the findings of this study are available from the corresponding author upon reasonable request.
