# Supplementary material for: Assignment-free chirality detection in unknown samples via microwave three-wave mixing
Source: Commun Chem. 2022 Mar 14;5:31. doi: 10.1038/s42004-022-00641-3 (PMC9814651; doi:10.1038/s42004-022-00641-3)
Supplement: Supplementary file 1 — Supplementary Information [file 42004_2022_641_MOESM1_ESM.pdf]

Supplementary Information

Supplementary Table 1 | List of three-wave mixing transitions for (R)-myrtenal, corresponding to Figure 2.

	J'	K_a'	K_c'	J"	K_a"	K_c"	Frequency/MHz	Transition type
	8	0	8	7	1	7	13661.8585	b
1	7	1	7	7	0	7	69.6411	c
	8	0	8	7	0	7	13731.4996	a
	8	0	8	7	1	7	13661.8585	b
2	8	1	8	8	0	8	38.0859	c
	8	1	8	7	1	7	13699.9444	a
	10	3	7	9	4	5	13833.8951	c
3	9	4	5	9	4	6	88.3310	a
	10	3	7	9	4	6	13921.8072	b
	6	3	4	5	2	3	14337.0904	b
4	6	3	3	6	3	4	93.6890	a
	6	3	3	5	2	3	14430.7794	c
	6	3	3	5	2	4	14901.5436	b
5	6	3	3	6	3	4	93.6690	a
	6	3	4	5	2	4	14807.8546	c
	10	1	9	9	2	8	17398.5173	b
6	10	2	9	10	1	9	248.7541	c
	10	2	9	9	2	8	17647.2711	a
	7	4	4	6	3	3	17904.4732	b
7	6	3	3	6	3	4	93.6280	a
	7	4	4	6	3	4	17998.1012	c
	7	4	3	6	3	4	18010.1251	b
8	6	3	3	6	3	4	93.6279	a
	7	4	3	6	3	3	17916.4972	c
	13	4	9	12	5	7	17895.9851	c
9	12	5	7	12	5	8	74.2297	a
	13	4	9	12	5	8	17970.2148	b

Supplementary Table 2 | Three-wave mixing transitions for (R)-1,2-propanediol, corresponding to Figure 3.

Species	J'	K_a'	K_c'	J"	K_a"	K_c"	Frequency/MHz	Transition type
conformer 1	2	2	1	2	1	1	14795.7831	c
	2	2	0	2	2	1	100.4028	a
	2	2	0	2	1	1	14896.1859	b
conformer 3	2	2	1	2	1	1	14797.7162	c
	2	2	0	2	2	1	95.1130	a
	2	2	0	2	1	1	14892.8290	b

Supplementary Table 3 | Three-wave mixing transitions for mixture of terpenes, corresponding to Figure 4

Species	J'	K_a'	K_c'	J"	K_a"	K_c"	Frequency/MHz	Transition type
β-pinene	7	1	7	6	0	6	16491.7295	b
	6	1	6	6	0	6	66.3070	c
	7	1	7	6	1	6	16425.4225	a
β-pinene	7	0	7	6	1	6	16392.0412	b
	6	1	6	6	0	6	66.2605	c
	7	0	7	6	0	6	16458.3015	a
fenchone	8	2	7	7	1	6	16492.5225	b
	8	2	7	8	1	7	26.6570	c
	8	1	7	7	1	6	16465.8655	a
carvone	15	1	15	14	0	14	17856.7875	b
	15	1	15	15	0	15	90.7070	c
	15	0	15	14	0	14	17766.0805	a
carvone	15	0	15	14	1	14	17641.1531	b
	15	1	15	15	0	15	90.4015	c
	15	1	15	14	1	14	17731.5545	a

Supplementary Note

Three-wave mixing spectra combine a non-linear detection method (M3WM) with broadband excitation. Even though we aimed for the highest sensitivity possible, we had to run quite underpowered given the broad fields, while keeping excitation via higher order harmonics in our chirp upconversion circuit minimal for better cancellation. Adding more chirp power (using amplifiers) increased the signal of enantiopure species but introduced non-linear components to the chirp that led to non-M3WM signals surviving cancellation. On the other hand, increasing the duration of the chirp was a cleaner way to increase the signal. Another factor that limited the overall sensitivity was that we did not want to introduce any asymmetries in the data acquisition process which limited our data acquisition velocity. Thus, optimal conditions for broadband M3WM involved the combination of controllability over phase and polarization with long and linear chirps.
